# Behavioral Phenotypes of Foxg1 Heterozygous Mice

**DOI:** 10.3389/fphar.2022.927296

**Published:** 2022-06-08

**Authors:** Skyler Younger, Sydney Boutros, Francesca Cargnin, Shin Jeon, Jae W. Lee, Soo-Kyung Lee, Jacob Raber

**Affiliations:** ^1^ Department of Behavioral Neuroscience, Oregon Health & Science University, Portland, OR, United States; ^2^ Spark Therapeutics, Inc., Philadelphia, PA, United States; ^3^ Department of Biological Sciences, University at Buffalo, Buffalo, NY, United States; ^4^ Department of Systems Pharmacology & Translational Therapeutics, Institute for Immunology, University of Pennsylvania, Philadelphia, PA, United States; ^5^ Departments of Neurology and Radiation Medicine, Division of Neuroscience, Oregon National Primate Research Center, Oregon Health & Science University, Portland, OR, United States

**Keywords:** open field, object recognition, wire hang, contextual fear memory, cued fear memory

## Abstract

FOXG1 syndrome (FS, aka a congenital variant of Rett syndrome) is a recently defined rare and devastating neurodevelopmental disorder characterized by various symptoms, including severe intellectual disability, autistic features, involuntary, and continuous jerky movements, feeding problems, sleep disturbances, seizures, irritability, and excessive crying. FS results from mutations in a single allele of the FOXG1 gene, leading to impaired FOXG1 function. Therefore, in establishing mouse models for FS, it is important to test if heterozygous (HET) mutation in the Foxg1 gene, mimicking genotypes of the human FS individuals, also manifests phenotypes similar to their symptoms. We analyzed HET mice with a null mutation allele in a single copy of Foxg1, and found that they show various phenotypes resembling the symptoms of the human FS individuals. These include increased anxiety in the open field as well as impairment in object recognition, motor coordination, and fear learning and contextual and cued fear memory. Our results suggest that Foxg1 HET mice recapitulate at least some symptoms of the human FS individuals.

## Introduction

FOXG1 is a member of the forkhead transcription factor family, which is one of the earliest transcription factors that begin to be expressed specifically in the neural progenitors of the forebrain (Tao and Lai, 1992). While roles of mouse Foxg1 in the development of forebrain as well as pathological conditions and symptoms caused by heterozygous (HET) mutations in the FOXG1 gene in humans have been relatively well-defined, behavioral consequences from mutations in the mouse Foxg1 gene just began to be understood. In this paper, we set out to systematically analyze behavioral phenotypes of Foxg1 HET mice, in which the Cre recombinase gene replaced a single copy of the Foxg1 gene, creating a Foxg1-Cre/+ mouse line (Hébert and McConnell, 2000).

In humans, mutations in a single allele of the *FOXG1* gene lead to FOXG1 syndrome (FS, aka a congenital variant of Rett syndrome) (Ehrhart et al., 2018; Srivastava et al., 2018; Vegas et al., 2018). Most FS patients carry *de novo* variants of the *FOXG1* gene, leading to impaired FOXG1 function. FS is a relatively newly defined rare and devastating neurodevelopmental disorder, and currently, over 1,000 patients have been identified (according to the FOXG1 Research Foundation), with numbers steadily increasing in recent years due to advancements in genetic testing. FS patients often present with severe brain structural deficits, including microcephaly, hypogenesis of the corpus callosum, and delayed myelination (Ehrhart et al., 2018; Srivastava et al., 2018; Vegas et al., 2018). Additionally, many patients experience epilepsy and cannot speak or walk on their own. FS is also characterized by autistic features, including repetitive movements, poor social interaction skills, including poor eye contact and a near absence of speech and language skills, and severe intellectual disability (Kortüm et al., 2011), and hence belongs to an autism spectrum disorder (ASD). Together, these clinical outcomes significantly impair FS patients, leaving them heavily dependent on caregivers throughout their lives. While current treatments for FS prioritize symptom management, there are no available options to treat the underlying causes of the condition.

In mice, complete knockout of the *Foxg1* gene results in a striking reduction of the cerebral hemispheres, which is likely due to reduced proliferation and precocious differentiation of Foxg1-deficient neural progenitors (Xuan et al., 1995; Hanashima et al., 2004). We have also reported roles of Foxg1 in post-mitotic neurons to generate the cortical laminar structure in an inside-out fashion and to form the corpus callosum (Cargnin et al., 2018). Several studies on Foxg1 HET mice, which mimic the genotypes of the human FS individuals, have been reported. Heterozygous deletion of *Foxg1* in mouse impairs neurogenesis in the postnatal hippocampus, particularly in the dentate gyrus (Shen et al., 2006). Foxg1 haploinsufficiency also leads to disrupted forebrain development, including a significant reduction in the volume of the neocortex, hippocampus and striatum (Eagleson et al., 2007; Cargnin et al., 2018). Foxg1 haploinsufficiency decreases the population of cortical intermediate progenitors *via* increased p21 expression, leading to thinner neocortices than wild-type controls, specifically in the supragranular layers (Siegenthaler et al., 2008; Cargnin et al., 2018; Pringsheim et al., 2019). Notably, Foxg1 HET mice show thinner neocortices than wild-type controls, mainly due to reduction of the supragranular layers. The axons of many supragranular neurons cross the midline of the brain and connect the two hemispheres, forming the corpus callosum (Siegenthaler et al., 2008; Cargnin et al., 2018). Consistently, we and others reported corpus callosum agenesis in Foxg1 HET brains (Eagleson et al., 2007; Cargnin et al., 2018; Pringsheim et al., 2019), a cardinal feature of the human KS brains (Ehrhart et al., 2018; Srivastava et al., 2018; Vegas et al., 2018).

Behavioral studies for Foxg1 HET mice have been only sporadic (Shen et al., 2006; Miyoshi et al., 2021). It is also important to note the differences in the mouse models used in the behavioral studies so far.

The current study involved HET mice in which a single copy of the intronless Foxg1 coding region was replaced with Cre. The Shen et al. study involved HET mice in which a single copy of the Foxg1 coding region was replaced with tetracycline transactivator (aTA) (Shen et al., 2006). A detailed description of the generation of model used for these behavioral studies was reported by Hanashima et al. (Hanashima et al., 2004). Behavioral phenotypes exhibited by the HET mice in this study were increased ambulation in a novel environment and impaired encoding or recognition of a fear-related context, consistent with dysfunction of the dorsal and ventral hippocampus or its efferents to the striatum (Fanselow, 2000). The Miyoshi et al. study involved HET mice in which a single copy of Foxg1 coding region was replaced with LacZ. Behavioral phenotypes in this model were impairments in social behavior and autism spectrum disorder ASD-related circuit (Miyoshi et al., 2021). In this paper, we set out to perform a series of behavioral assays with the Foxg1-Cre/+ mouse line (Hébert and McConnell, 2000), and find several phenotypes that recapitulate at least some symptoms of the human FS individuals. Overall, our results validate Foxg1 HET mice as an animal model to recapitulate at least some symptoms of the human FS individuals.

## Materials and Methods

### Mice

The Cre recombinase gene replaced the FoxG1 gene, creating a Foxg1-Cre/+ mouse line. Foxg1-Cre/+ mice on a C57Bl/6J background were bred to create Foxg1-Cre/+ and Foxg1-+/+ (wild-type) littermates. Male and female wild-type and Foxg1-Cre/+ littermates (*n* = 36 total) were genotyped and used in this study. Mice were on average 50 days of age at the beginning of behavioral testing (wild-type males: 49.2 ± 0.6; wild-type females: 50.6 ± 0.5; Foxg1 males: 50.1 ± 0.6; Foxg1females: 53.1 ± 0.5). The Foxg1 mutant (*n* = 9 male and *n* = 9 female mice) and wild-type (*n* = 7 male and *n* = 11 female mice) littermates were tested for exploratory behavior in the open field (week 1: Monday-Wednesday), object recognition (week 1: Thursday-Friday), muscle strength and condition in the wire hang test (week 2: Monday-Tuesday), activity and spontaneous alteration in the Y maze (week 2: Thursday-Friday), and hippocampus-dependent contextual fear memory and hippocampus-independent cued fear memory (week 3: Monday-Wednesday) as described in detail below. All mice were singly housed starting 4 days prior to testing. Food and water were provided *ad libitum* and lights were on a standard 12 h light: dark cycle. All animal procedures were reviewed and approved by the OHSU IACUC and in accordance with AAALAC standards. All procedures followed the ARRIVE guidelines. Researchers were blinded to genotype throughout the duration of experiments. Mice were singly housed during testing.

### Open Field and Object Recognition

To assess locomotion, spatial habituation learning, and anxiety-like behaviors, mice were placed into an open field (41 × 41 cm) and allowed to explore for 5 min over three subsequent days, as described (Torres et al., 2018). The enclosures were thoroughly cleaned with 0.5% acetic acid and dried between each trial. Light intensity in the enclosures ranged from 300 to 500 lux. Animals were video recorded at a rate of 15 samples per second, and total distance moved, average velocity, and time spent in the center (defined as a center square sized 20 × 20 cm) were analyzed using Ethovision XT 7.1 software (Noldus Information Technology, Wageningen, Netherlands).

Following open field testing, two similar objects (orange hexagonal prism blocks) were secured in place in the center of the open field. Animals were allowed to explore for 15 min. The next day, one object was replaced with a distinct, novel object (green triangular block). Again, animals were allowed to explore for 15 min. Total time exploring the objects was manually recorded for both sessions, as well as time spent exploring each object. The researcher recording exploration time was blinded to the groups. To assess object recognition, the discrimination index on day 2 (time spent with novel object-time spent with familiar object)/(time spent with both objects) was calculated for each mouse.

### Wire Hang Test

Motor function was also assessed using the wire hang task, adopting the “falls and reaches” method described by van Putten (Van Putten et al., 2016). A 55-cm wide 2-mm thick metallic wire was secured to two vertical stands. Mice were placed on the wire so that they were hanging only by their front paws. Mice started with a fall score of 10 and a reach score of 0. Over the duration of 180 s, mice lost one point every time they fell and gained one point every time they reached one of the poles holding up the wire. The time of each fall or reach event was also recorded. Each time a mouse fell or reached, the timer was paused to replace the mouse in the center of the wire again. This test assesses endurance and strength, along with complex motor coordination.

### Y Maze

Activity levels and hippocampus-dependent spontaneous alternations were assessed in the Y-maze. The Y-shaped maze (O’ Hara & Co., Ltd., Tokyo, Japan) has raised sides (3.8 cm bottom width, 12.55 cm top width, 12.55 cm height) with plastic, opaque grey arms (37.98 cm length) at a 120 angle from each other. Mice were placed in the center of the maze at the beginning of a 5-min trial. The maze was cleaned with 0.5% acetic acid between trials. Performance of the mice was recorded using Ethovision 15 XT software, Noldus Information Technology, Wageningen, The Netherlands. Digital videos were later analyzed using hand scoring to measure the number of arm entries and to calculate the percent spontaneous alternations, a cognitive performance measure based on the innate tendency of rodents to explore a prior unexplored arm the Y-maze. The criteria for an arm entry were when all four limbs were within the arm. The spontaneous alternation percentage was calculated by dividing the number of 3-arm alternations by the number of possible 3-arm alternations and multiplying the value by 100. Mice with intact working memory should show a high percentage of spontaneous alterations, suggesting that they recognized previously explored arms within a short period of time. Mice with impaired working memory will repeatedly re-enter the same arms without exploring the others, suggesting impairments in short-term memory.

### Fear Conditioning

Mice were tested for contextual and cued fear learning and memory, as described (Olsen et al., 2012). Briefly, mice were placed into a sound-attenuating chamber. After a 120s baseline period without any stimuli, a tone (80 dB, 2,800 Hz) was played for 30s, which co-terminated with a 2 s shock (0.5 mA). This was followed by a 120s period with no stimuli before the tone-shock pairing was repeated again. In total, mice received 2 tone-shock pairings. The chambers were thoroughly cleaned with 0.5% acetic acid between animals. Twenty-4 hours later, mice were tested for contextual and cued fear memory recall. For contextual memory recall, mice were placed into the same chamber for a period of 5 min, with no cues presented. For cued memory recall, assessed 3 h later, the chambers were changed to remove any contextual cues (floors were covered with a solid, white panel, the roof and walls were changed to a black triangle, there was a vanilla scent, and a 10% isopropanol solution was used for cleaning). Animals had a baseline period of 90s with no stimuli; at 90s, the same tone played for a total of 3 min.

All trials were recorded and analyzed with Video Freeze software (PMED-VFC-NIR-M, Med Associates, Inc. Fairfax, VT) and automatic outputs were generated to analyze average motion and time freezing (defined as a minimum of 30 frames, or 1s, of total movement cessation). Percent time freezing was analyzed as measure of fear memory.

### Statistical Analyses

All data are expressed as mean ± standard error of the mean. The data were analyzed using SPSS 24 software (IBM, Armonk, NY) and GraphPad 6 (Prism, San Diego, CA). Two-way ANOVAs were run using genotype and sex as between-subject variables. In the cases where sex was determined to not be significant, data were analyzed with a univariate ANOVA with genotype as the between-group variable and the sexes collapsed. A repeated-measures ANOVA was used to analyze activity in the open field over days and for fear acquisition-related measures. Unless indicated that Sidak or 2-tailed t tests were used, all statistical effects indicated are main effects. Results were considered significant at a *p* value smaller than 0.05.

## Results

### Foxg1 Heterozygous Mice Show Increased Activity Levels and Increased Measures of Anxiety in the Open Field and Impaired Object Recognition

**FIGURE 1:**
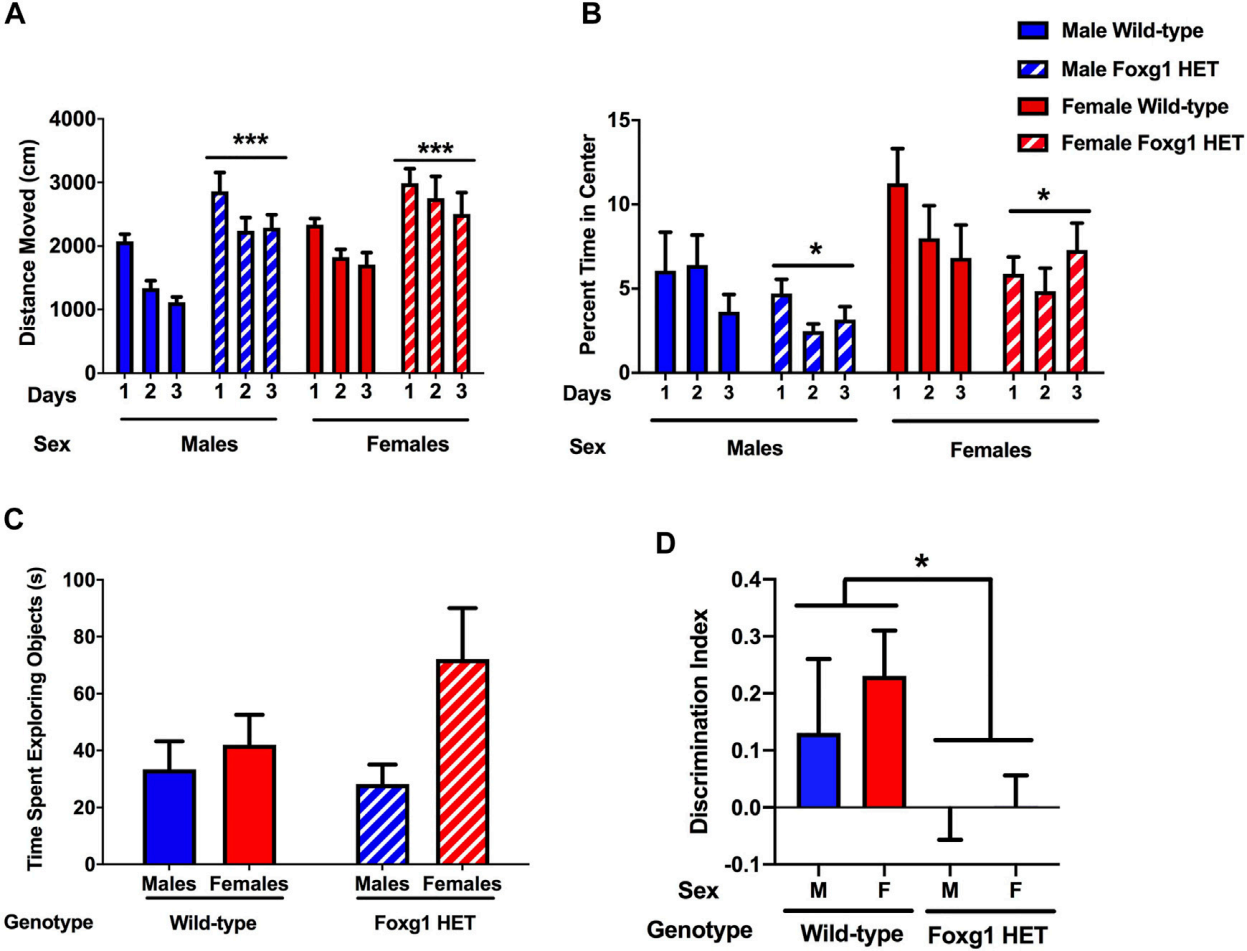
Performance in the open field and object recognition of Foxg1 HET and wild-type mice. **(A)** Activity levels in the open field. Foxg1 HET mice showed higher activity levels than wild-type mice. Effect of genotype: ***p < 0.001. The mice showed spatial habituation, with activity in the open field decreasing over the 3 days [*F* (1.378, 44.10) = 11.89; *p* = 0.0004]. **(B)** Anxiety levels in the open field. Foxg1 HET mice spent less time in the more anxiety-provoking center of the open field than wild-type mice. Effect of genotype: *p < 0.05. Females spent more time in the center of the open field than males (*p* < 0.05). **(C)** There was no genotype difference in the time the mice spent exploring both object in the object recognition test. There was an effect of sex with the female mice exploring the objects more than the male mice that was mostly driven by Foxg1 HET female mice (*p* < 0.05). **(D)** The discrimination index was greater in wild-type mice than Foxg1 HET mice. Effect of genotype: *p = 0.0188.

To assess locomotion, spatial habituation learning, and anxiety-like behaviors, mice were placed into an open field and allowed to explore for 5 min over three subsequent days, as described (Torres et al., 2018). In the open field, the mice were placed in the enclosure for 5 min on three subsequent days. There was an overall effect of genotype [*F* (1, 32) = 22.98, *p* < 0.001] on activity levels across days with higher activity levels in Foxg1 HET than wild-type mice (Figure 1A). There was no effect of sex or sex × genotype interaction. The mice showed spatial habituation, as activity in the open field decreased over the 3 days [*F* (1.378, 44.10) = 11.89; *p* = 0.0004].

The time spent in the center of the open field over the 3 days was analyzed to assess anxiety levels. There was an effect of genotype [*F* (1, 32) = 4.206, *p* = 0.049], with Foxg1 HET spending less time in the center of the open field than wild-type mice across days, suggesting increased anxiety levels in the Foxg1 HET mice (Figure 1B). This is remarkable considering that the overall activity levels were higher in the Foxg1 HET than wild-type mice. There was also an effect of sex for time spent in the center of the open field [*F* (1, 32) = 6.84, *p* = 0.014], with females spending more time in the center of the open field than males. There was no sex × genotype interaction.

Next, object recognition was assessed. On day 4, the mice were introduced to two identical objects. On day 5, one of the objects that was present on day 4 was replaced by a novel one. There was no genotype difference in the time the mice spent exploring both object [*F* (1, 32) = 1.02; *p* = 0.302] (Figure 1C). There was an effect of sex with the female mice exploring the objects more than the male mice [*F* (1, 32) = 4.532; *p* = 0.0411] but that seemed driven by the Foxg1 HET female mice (Figure 1C). The wild-type mice showed object recognition and had a greater discrimination index than Foxg1 HET mice (Figure 1D). In addition, the discrimination index of Foxg1 HET mice was not different from 0.

### Impaired Performance of Foxg1 Heterozygous Mice in the Wire Hang Test

**FIGURE 2:**
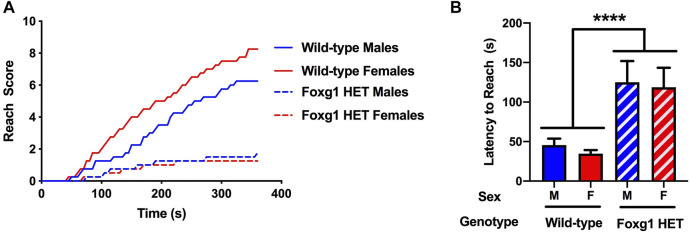
Performance of Foxg1 HET and wild-type mice in the wire hang test. **(A)** Foxg1 HET mice had lower reach scores than wild-type mice [*F* (1, 28) = 14.71, *p* = 0.001, repeated measures]. There was also a genotype X sex × time interaction [*F* (36, 1,008) = 1.91, *p* = 0.001] and a genotype × time interaction [*F* (36, 1,008) = 13,20, *p* < 0.001]. Reach scores were higher in wild-type females than males. No sex differences in reach scores were seen in Foxg1 HET mice. **(B)** The latency to reach was greater in Foxg1 HET than wild-type mice. Effect of genotype: ****p < 0.001.

Next, motor function was assessed using the wire hang task, adopting the “falls and reaches” method described by van Putten (Van Putten et al., 2016). For the reach score in the wire hang test, there was an effect of genotype [*F* (1, 28) = 14.71, *p* = 0.001, repeated measures], with lower reach scores in Foxg1 HET than wild-type mice (Figure 2A). There was also a genotype x sex × time interaction [*F* (36, 1,008) = 1.91, *p* = 0.001] and a genotype × time interaction [*F* (36, 1,008) = 13,20, *p* < 0.001]. While the reach scores in Foxg1 HET females and males were similar, reach scores were higher in wild-type females than males (Figure 2A). In addition, reach scores of Foxg1 HET mice improved much less with training and reached a plateau below 2. In addition, the latency to reach was greater in Foxg1 HET than wild-type mice (Figure 2B). There was no effect of sex or sex × genotype interaction.

### Foxg1 Heterozygous Mice Show Increased Activity Levels in the Y Maze

**FIGURE 3:**
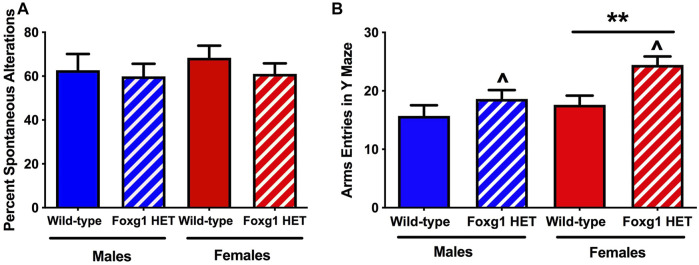
Performance of Foxg1 HET and wild-type mice in the Y maze test. **(A)** There was no genotype difference in spontaneous alternation in the Y maze. **(B)** The number of arm entries was higher in Foxg1 HET than wild-type mice. Effect of genotype: ^*p* < 0.05. In addition, female Foxg1 mutant mice entered more arms in the Y maze than sex-matched wild-type mice. Effect of genotype in female mice only: **p = 0.0057. In contrast, no genotype difference in arm entries in the Y maze was seen in male mice.

To assess activity levels and hippocampus-dependent spontaneous alternations, we employed the Y-maze (O’ Hara & Co., Ltd., Tokyo, Japan). There was no genotype difference in spontaneous alternation in the Y maze (Figure 3A). Consistent with the activity levels seen in the open field, the number of arm entries were higher in Foxg1 than wild-type mice (*t* = 2.639, *p* = 0.0125, 2-tailed *t*-test) (Figure 3B). This genotype difference was driven by the female mice. Similar to the pattern seen for time spent exploring both objects, female Foxg1 mutant mice entered more arms in the Y maze than sex-matched wild-type mice (*t* = 3.165, *p* = 0.0057, 2-tailed *t-*test). In contrast, no genotype difference in arm entries in the Y maze was seen in male mice.

### Foxg1 Heterozygous Mice Show an Increased Shock Response but Impaired Fear Learning and Impaired Contextual and Cued Fear Memory

**FIGURE 4:**
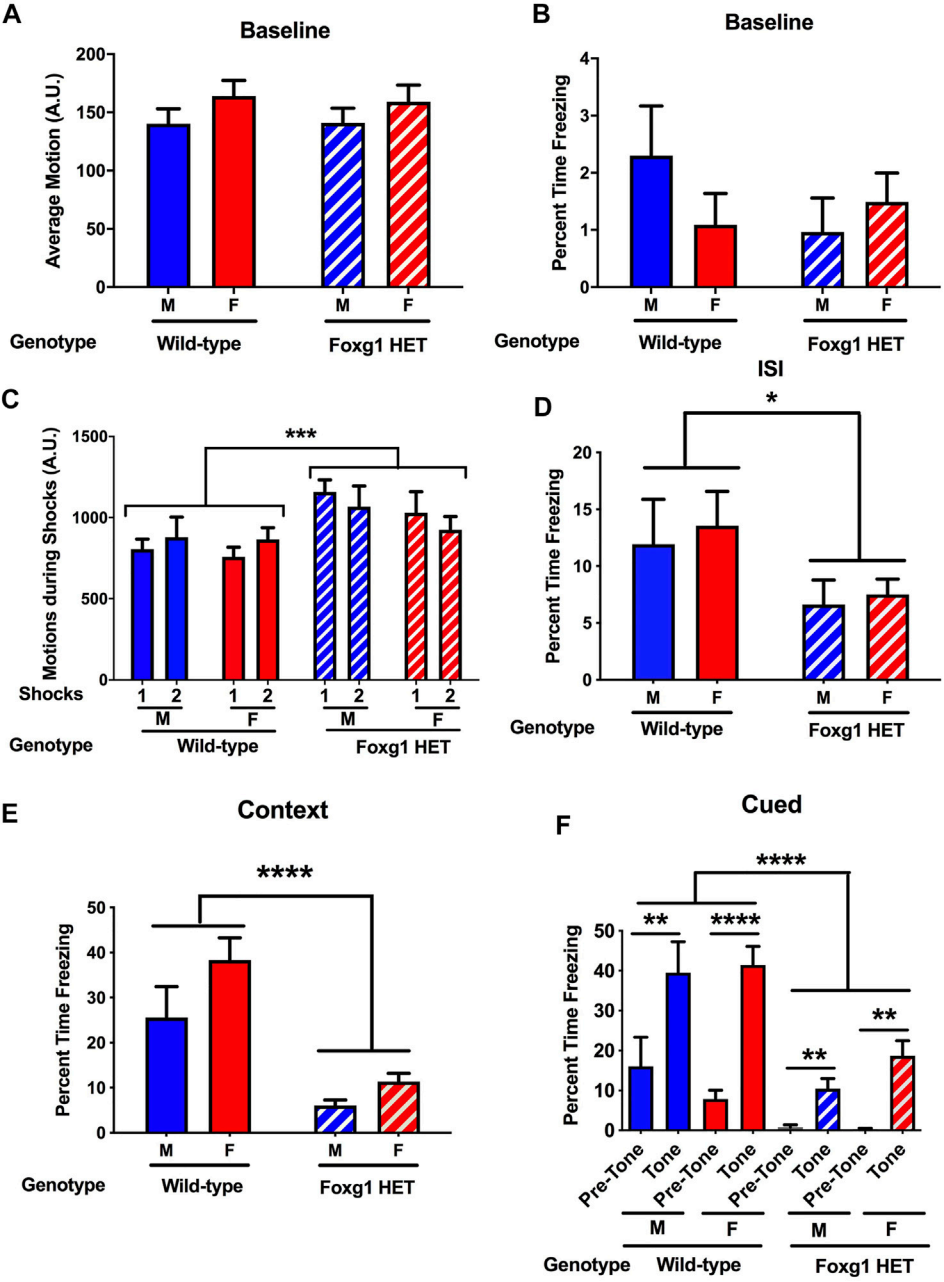
Fear learning and memory of Foxg1 HET and wild-type mice. **(A)** There was no genotype difference in activity levels during the baseline period (prior to the first tone). **(B)** There was no genotype difference in percent freezing during the baseline period. **(C)** Foxg1 HET mice showed an increased response to the shocks (average motion during the shocks). Effect of genotype: ***p < 0.0001. **(D)** Foxg1 HET mice showed reduced freezing between the two tone-shock intervals. Effect of genotype: *p < 0.05. **(E)** Foxg1 HET mice showed reduced freezing in the contextual fear memory test. Effect of genotype: ****p < 0.0001. **(F)** Across the baseline and tone periods, Foxg1 HET mice froze less than wild-type mice. Effect of genotype: ****p < 0.0001. Wild-type male mice and Foxg1 HET and wild-type female mice froze more during the tone than during the pre-tone. **p < 0.01; ****p < 0.001 pre-tone versus tone periods, Sidak tests.

Finally, mice were tested for freezing during the contextual and cued fear memory tests (Olsen et al., 2012). On the training day, there was no genotype difference in activity (Figure 4A) or percent freezing (Figure 4B) during the baseline period (prior to the first tone). The mice received two tones, which co-terminated with shocks. Foxg1 HET mice showed an increased response to the shocks average motion during the shocks [*F* (36, 36) = 3.824, *p* < 0.0001] (Figure 4C). In contrast, Foxg1 HET mice showed reduced freezing between the two tone-shock interval, indicating reduced acquisition of fear (*t* = 2.212, *p* = 0.018, 2-tailed *t*-test) (Figure 4D).

The following day, hippocampus-dependent contextual fear memory was assessed by placing the animals back in the same environment (context) as that present during training. Foxg1 HET mice showed dramatically reduced freezing in the contextual fear memory test (*F* = 11.54, *p* < 0.0001, 2-tailed *t*-test) (Figure 4E).

Subsequently, the mice were placed in a novel environment to assess hippocampus-independent cued fear memory. Following a baseline period, the mice were exposed to the tone that co-terminated with a shock during the training day. Across the pre-tone and tone periods, Foxg1 HET mice froze less than wild-type mice (Figure 4F) (Effect of genotype: *F* = 14.30, *p* < 0.0001). Wild-type male (*t* = 3.657, *p* < 0.01, Sidak test) mice and wild-type (*t* = 6.566, *p* < 0.001, Sidak test) and Foxg1 HET (*t* = 3.249, *p* < 0.01, Sidak test) female mice froze more during the tone than during the pre-tone, indicating that they both showed cued fear memory. As the Foxg1 HET mice showed less freezing during acquisition of fear on the training day, impaired fear learning might have contributed to the reduced freezing levels of Foxg1 HET mice during the contextual and cued fear memory tests.

## Discussion

This study represents a systematic assessment of behavioral phenotypes of Foxg1 HET mice, and our results reveal profound phenotypes of Foxg1 HET mice. They include increased activity levels in the open field and Y maze tests, increased anxiety levels in the open field and enhanced responsiveness to shock during fear learning, impaired hippocampus-dependent object recognition, fear learning and hippocampus-dependent contextual fear learning and impaired hippocampus-independent cued fear memory, and impaired motor function in the wire hang test. Overall, our behavioral studies are aligned with the prior studies with Foxg1 HET mice (Shen et al., 2006; Miyoshi et al., 2021) while some specific differences are also notable. As in our study, hyperactivity and impaired contextual fear memory were seen in Foxg1 HET mice (Shen et al., 2006). In that study, the pre-tone and tone freezing in the cued fear memory test seemed also lower in the Foxg1 HET than wild-type mice. In addition, the levels of freezing of Foxg1 HET mice in the contextual fear memory test were even more profound and not different for freezing levels during the baseline period prior to the first tone during fear learning. In our study, the freezing levels of the Foxg1 HET mice during the contextual fear memory test were higher than those during the baseline period prior to the first tone during fear learning (*t* = 5.725, *p* < 0.0001, 2-tailed *t*-test). It is hard to compare those results as the age of the mice tested in that study did not seem indicated. However, no alterations in activity levels and slightly decreased anxiety levels were seen in 5-week-old Foxg1 HET mice (Miyoshi et al., 2021). As distinct mouse models were used in the behavioral studies, it is conceivable that this might have contributed to some of the divergent finding. In addition, as in the current study mice were tested starting at 50 days of age, these data indicate that in Foxg1 mice behavioral phenotypes, including a phenotype showing enhanced anxiety levels, might be revealed with age when compensatory mechanisms might be unable to rescue them. As measures of anxiety in current study were assessed in the open field and in the Miyoshi et al. study in the elevated plus maze (Miyoshi et al., 2021), we cannot exclude that differences in these two behavioral tests might have contribute to these divergent findings. For example, reduced anxiety levels in mice lacking the histamine H3 receptor, reduced anxiety levels were seen in the elevated plus maze but not in the open field (Rizk et al., 2004). Reduced anxiety levels in the elevated plus maze without altered anxiety levels in the open field was also seen in mice lacking proper glucocorticoid dimerization (van Looveren et al., 2019), supporting that these two tests measure different anxiety measures. Importantly, the phenotypes we observed in this paper recapitulate some symptoms of human FS individuals, supporting the translational value of this mouse model for the development and testing of therapeutic strategies.

Typically, activity levels are positively related to times spent in the center of open field (Sestakova et al., 2013). However, in Foxg1 HET mice enhanced activity levels and enhanced anxiety levels are seen in the open field. These data indicate that the enhanced anxiety levels seen are not the result of potential motor problems. Consistent with enhanced anxiety levels, the response to the shocks in the fear conditioning test was enhanced in Foxg1 HET mice. However, although the Foxg1 HET mice showed this enhanced response, the freezing during the tone-shock intervals, a measure of fear learning, was impaired. In contrast to fear learning, spatial working memory in the Y maze was not affected in Foxg1 HET mice. These data indicate that the fear learning impairment is not reflecting a general learning impairment. As the brain regions involved in fear learning are well worked out (Quirk and Beer, 2006; Raber et al., 2019), this will further facilitate assessing the anatomical specificity of the Foxg1 HET phenotypes.

Object recognition memory involving a 24-h delay between learning and memory involves the hippocampus (Ennaceur, 2010; Raber, 2015; Boutros et al., 2021). Contextual fear memory is also hippocampus-dependent (Maren et al., 2013; Raber et al., 2013). As both were affected in the Foxg1 HET mice, these data suggest that the hippocampus might be especially vulnerable. The enhanced anxiety levels might also be related to the hippocampus (Baksh et al., 2021) (Revest et al., 2009). In this regard, it is notable that hippocampal structural anomaly, particularly with hypogenesis of the hippocampal dentate gyrus was reported for Foxg1 mutant mice along with corpus callosum agenesis and microcephaly (Shen et al., 2006; Tian et al., 2012; Cargnin et al., 2018). Also, hippocampus atrophy has also been reported in FS cases (Harada et al., 2018).

In the cued fear memory test, the Foxg1 HET mice freeze much less prior to and during the tone than wild-type mice. As there are no genotype differences in freezing during the baseline period prior to the first tone on the training day, these data might suggest that the Foxg1 HET mice might have no problem in distinguishing the novel environment in the cued fear memory test from the familiar environment during fear training the contextual fear memory test. The fact that the Foxg1 HET mice freeze more during the tone than prior to the tone indicates that Foxg1 HET mice do have associative learning and recall the pairing of the tone with the shock during the training. The percent reduction in freezing in Foxg1 HET mice as compared to wild-type mice during the ISI seems less profound (around 50%) than the genotype difference in freezing seen during the contextual fear memory (around 66%) and during the tone in the cued fear memory test (around 66%). This suggests that in Foxg1 HET mice the memory problem might be more profound than the learning problem. As in contrast to object recognition and contextual and cued fear memory spatial working memory in the Y maze was not affected, future efforts are warranted to assess whether Foxg1 HET mice show intact memory at shorter delays between learning and memory and whether impaired memory is associated with alterations in specific molecular measures in the hippocampus.

The current study reveals the importance of including both females and males in assessing behavioral phenotypes related to Foxg1 HET. The sex differences in female mice spending more time in the more anxiety-provoking center of the open field and being more active by entering more arms in the Y maze were driven by the Foxg1 HET female mice. In addition, while a sex difference in reach scores in the wire hang test with greater scores in females than males was seen in wild-type mice, no sex difference in reach scores was seen in Foxg1 HET mice. These data highlight the need to carefully consider sex differences and include sufficient females and males, both patients and controls, in human clinical studies. Five female and six male patients were included in a study of only eleven patients with a 2–31 age range, while 14 female and one male patients were included in earlier studies with a 10 months to 22 age range (Kortüm et al., 2011). A larger effort involving 50 patients is ongoing but depending on the age range it might still be challenging to reveal potential sex differences in the behavioral phenotypes.

Notably, with more patients being diagnosed as FS, it became clear that the spectrum of symptoms widely differs among patients depending on the type and location of the mutation in the *FOXG1* gene (Mitter et al., 2018; Vegas et al., 2018), highlighting the need for patient-specific animal models and personalized therapeutic intervention for FS patients. The existing Foxg1-null mouse lines, including the Foxg1-Cre/+ mouse line utilized in the current studies (Xuan et al., 1995; Hébert and McConnell, 2000; Shen et al., 2006; Miyoshi and Fishell, 2012), might be inadequate to fully address this critical issue. For instance, while some FS patients show hyperactivity, many FS patients show hypoactivity, unlike the hyperactivity reported for Foxg1 HET mice as reported in this study and by Shen et al. (Shen et al., 2006). Preliminary results with our patient-specific mouse models reveal faithful recapitulation of the locomotion activity of corresponding patients with either no changes in motor activity, hyperactivity, or hypoactivity.

## Data Availability

The raw data supporting the conclusions of this article will be made available by the authors upon request, without undue reservation.
